# Supplementary material for: Antibody-Mediated In Vitro Activation and Expansion of Blood Donor-Derived Natural Killer Cells with Transient Anti-Tumor Efficacy
Source: Biomedicines. 2025 Nov 29;13(12):2934. doi: 10.3390/biomedicines13122934 (PMC12730846; doi:10.3390/biomedicines13122934)
Supplement: Supplementary file 1 [file biomedicines-13-02934-s001.zip › biomedicines-3892949-supplementary.pdf]

Supplementary Materials

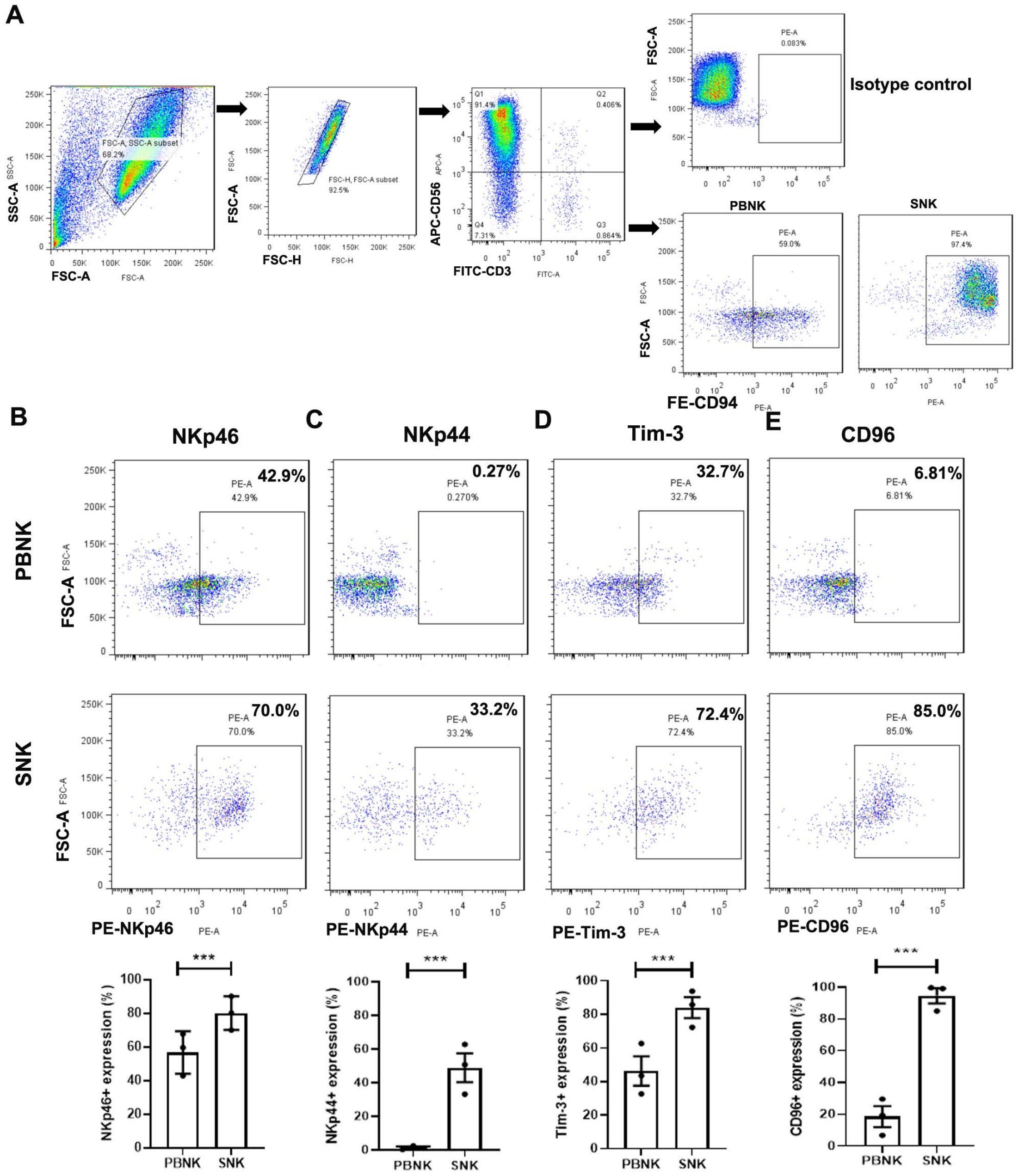

Figure S1. SNK cells exhibit higher expression levels of activation and inhibitory receptors compared with PBNK cells. (**A**) The gating strategy for NK cells ($CD3^{-}$ $CD56^{+}$) and activation and inhibitory receptors. Frequency of NKp46 (**B**), NKp44 (**C**), Tim-3 (**D**), and CD96 (**E**) was determined by flow cytometry, respectively. Data are shown as a mean ± SEM. P values are analyzed with unpaired Student's *t* test.

Statistically significant differences are shown with asterisks (**, $P<0.01$ and ***, $P<0.001$).

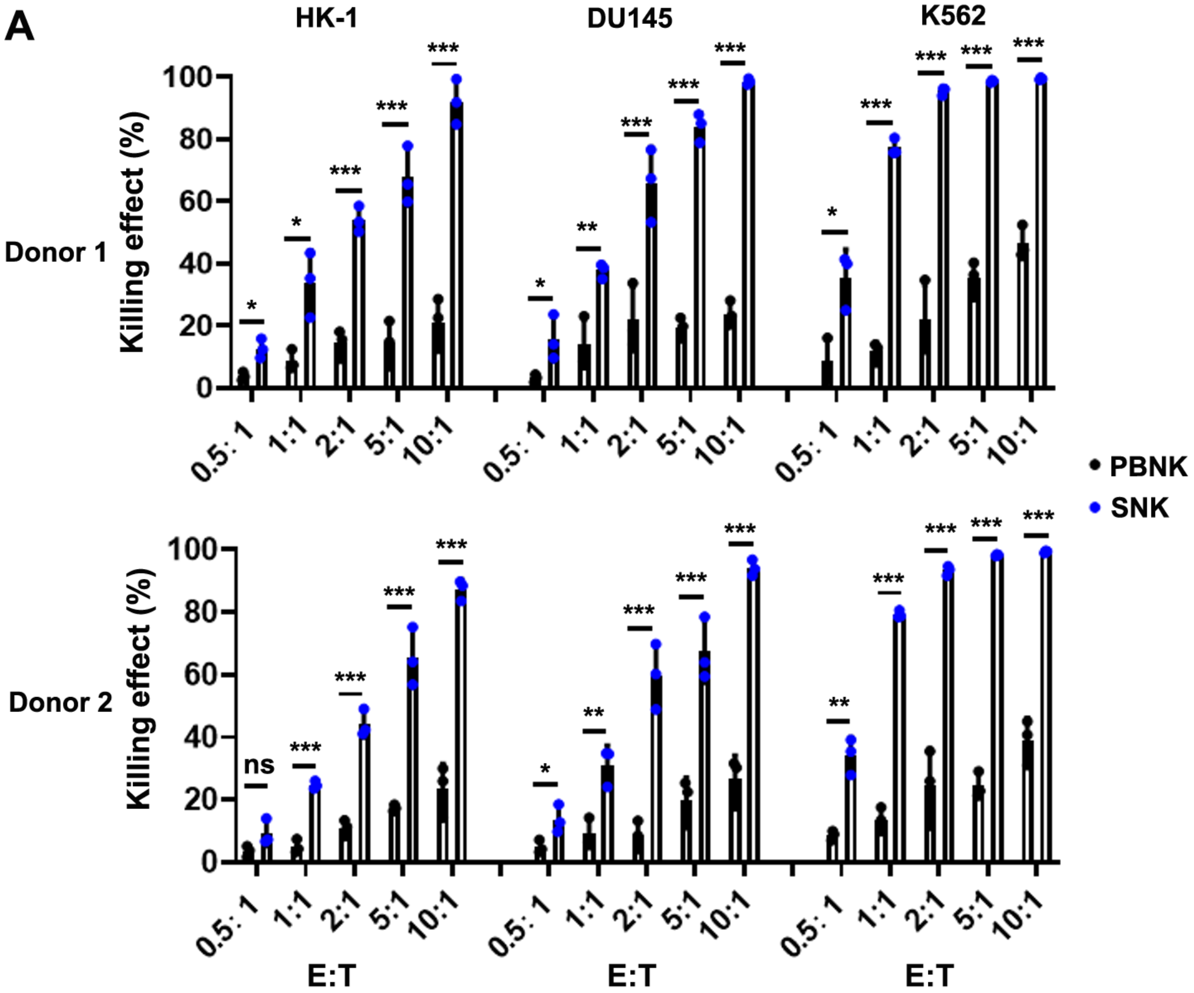

Figure S2. SNK cells of donor donor 1 and donor 2 kill HK-1, DU145, and K562 tumor cells, related to Figure 3. (**A**) SNK cells of indicated donors were incubated with Luc-expressing target cell lines for 6 h, after which specific cytolysis was determined by luciferase measurement. Mean values ± SEM of triplicate cultures are shown. Statistical analysis was performed using One-way ANOVA and Student's *t* test. Significant differences are indicated as *$P < 0.05$, ** $P < 0.01$, and ***$P < 0.001$; ns = not significant.

Table S1. Antibodies used in flow cytometry.

Fluorochrome/Marker	Catalog No	Source	Dilution
FITC-CD3	555332	BD Pharmingen	1:100
APC-CD56	555518	BD Pharmingen	1:100
PE-CD69	985202	BioLegend	5μL/test
PE-CD94	305506	BioLegend	5μL/test
PE-NKG2D	320805	BioLegend	5μL/test
PE-NKG2C	375003	BioLegend	5μL/test
PE-NKp46	331907	BioLegend	5 μL/test
PE-NKp44	325107	BioLegend	5μL/test
PE-Tim-3	364806	BioLegend	5μL/test
PE-CD96	338406	BioLegend	5μL/test
